# Dopamine D1-like receptor signalling in the hippocampus and amygdala modulates the acquisition of contextual fear conditioning

**DOI:** 10.1007/s00213-015-3897-y

**Published:** 2015-03-07

**Authors:** Florence C. Heath, Regimantas Jurkus, Tobias Bast, Marie A. Pezze, Jonathan L. C. Lee, J. Peter Voigt, Carl W. Stevenson

**Affiliations:** 1 School of Biosciences, University of Nottingham, Sutton Bonington Campus, Loughborough, LE12 5RD UK; 2 School of Mathematical Sciences, University of Nottingham, University Park, Nottingham, NG7 2RD UK; 3 School of Psychology and Neuroscience, University of Nottingham, University Park, Nottingham, NG7 2RD UK; 4 School of Psychology, University of Birmingham, Edgbaston, Birmingham, B15 2TT UK; 5 School of Veterinary Medicine and Science, University of Nottingham, Sutton Bonington Campus, Loughborough, LE12 5RD UK

**Keywords:** Amygdala, Contextual fear conditioning, Dopamine, D1 receptor, Hippocampus, Locomotor activity, Memory, Retrieval, Reconsolidation, Shock sensitivity

## Abstract

**Rationale:**

Dopamine D1-like receptor signalling is involved in contextual fear conditioning, but the brain regions involved and its role in other contextual fear memory processes remain unclear.

**Objectives:**

The objective of this study was to investigate (1) the effects of SCH 23390, a dopamine D1/D5 receptor antagonist, on contextual fear memory encoding, retrieval and reconsolidation, and (2) if the effects of SCH 23390 on conditioning involve the dorsal hippocampus (DH) and/or basolateral amygdala (BLA).

**Methods:**

Rats were used to examine the effects of systemically administering SCH 23390 on the acquisition, consolidation, retrieval and reconsolidation of contextual fear memory, and on locomotor activity and shock sensitivity. We also determined the effects of MK-801, an NMDA receptor antagonist, on contextual fear memory reconsolidation. The effects of infusing SCH 23390 locally into DH or BLA on contextual fear conditioning and locomotor activity were also examined.

**Results:**

Systemic administration of SCH 23390 impaired contextual fear conditioning but had no effects on fear memory consolidation, retrieval or reconsolidation. MK-801 was found to impair reconsolidation, suggesting that the behavioural parameters used allowed for the pharmacological disruption of memory reconsolidation. The effects of SCH 23390 on conditioning were unlikely the result of any lasting drug effects on locomotor activity at memory test or any acute drug effects on shock sensitivity during conditioning. SCH 23390 infused into either DH or BLA impaired contextual fear conditioning and decreased locomotor activity.

**Conclusions:**

These findings suggest that dopamine D1-like receptor signalling in DH and BLA contributes to the acquisition of contextual fear memory.

## Introduction

The neurotransmitter dopamine plays a crucial role in memory processing. Although well known for its involvement in appetitive learning and memory (Schultz 2013), dopamine also mediates certain aversive memory processes (Pezze and Feldon 2004; Iordanova 2009; Volman et al. 2013). During Pavlovian fear conditioning, an innocuous conditioned stimulus (CS) is paired with an aversive unconditioned stimulus (US); this results in the CS becoming associated with the US, such that the CS alone elicits fear responding after conditioning (Fendt and Fanselow 1999). Following conditioning, fear associated with the CS is transferred to long-term memory through consolidation (McGaugh 2000). Upon retrieval, fear memory can become labile which may allow for the updating of the memory to maintain its relevance, before it is restabilized through reconsolidation (Lee 2009).

Dopamine D1-like receptor signalling is involved in Pavlovian fear conditioning to discrete and contextual cues. SCH 23390 is a selective D1/D5 receptor antagonist in vivo (Bourne 2001) that has been used extensively as a pharmacological tool for investigating the role of D1-like receptor signalling in modulating fear memory processing. Systemic administration of SCH 23390 before training impairs fear-potentiated startle and contextual fear conditioning (Inoue et al. 2000; Greba and Kokkinidis 2000; Calzavara et al. 2009). In contrast, SCH 23390 administered immediately after training has no effect on contextual fear conditioning (Inoue et al. 2000; Bai et al. 2009), suggesting that D1/D5 receptor antagonism impairs the acquisition, but not consolidation, of contextual fear. Yet the brain regions involved in mediating the effects of SCH 23390 on contextual fear conditioning remain unclear. The dorsal hippocampus (DH) and basolateral amygdala (BLA) are both well known for their involvement in contextual fear conditioning. One prevalent view is that contextual representations are encoded by DH and associated with the US in BLA (Anagnostaras et al. 2001). Both DH and BLA receive dopamine projections from the ventral tegmental area (VTA) and show D1/D5 receptor expression (Huang et al. 1992; Scibilia et al. 1992; Gasbarri et al. 1997; Pinard et al. 2008; Muller et al. 2009). SCH 23390 suppresses long-term potentiation in DH and BLA, suggesting that D1-like receptor signalling is involved in synaptic plasticity in these regions (Otmakhova and Lisman 1996; Huang and Kandel 1995, 2007). Infusion of SCH 23390 into DH impairs spatial learning, and SCH 23390 infusion into BLA impairs fear learning to discrete cues (Greba and Kokkinidis 2000; O’Carroll et al. 2006; Pezze and Bast 2012). However, the role of D1-like receptor signalling in these areas in regulating contextual fear conditioning is poorly understood; therefore, one of the aims of the present study was to address this issue.

More recent studies have begun to investigate the role of dopamine transmission in memory reconsolidation. Amphetamine, an indirect dopamine receptor agonist, enhances the reconsolidation of conditioned place preference, but not Pavlovian conditioned approach, memory (Blaiss and Janak 2006, 2007). D3 receptor antagonism interferes with the reconsolidation of drug-associated memory (Yan et al. 2013, 2014). SCH 23390 also disrupts drug-associated memory reconsolidation and the reconsolidation of passive avoidance memory (Sherry et al. 2005; Yan et al. 2014). Infusion of SCH 23390 into medial prefrontal cortex impairs the reconsolidation of recognition memory under certain conditions (Maroun and Akirav 2009). However, the potential involvement of D1-like receptor signalling in modulating the reconsolidation of contextual fear memory has not been examined.

Here we sought to confirm and extend previous findings by examining (1) the effects of SCH 23390 on the acquisition, consolidation, retrieval and reconsolidation of contextual fear memory, (2) if any effects of SCH 23390 on conditioning are attributable to non-specific drug effects on locomotion or nociception, and (3) the effects of infusing SCH 23390 into DH or BLA on contextual fear conditioning and locomotion.

## Methods

### Animals

Male Lister hooded rats (250–400 g; Harlan, UK, or Charles River, UK) were group-housed on a 12-h light/dark cycle (lights on at 0800) and had free access to food and water. The principles of laboratory animal care were followed, and all experimental protocols were performed in accordance with internal ethical review and the Animals (Scientific Procedures) Act 1986, UK. All behavioural testing occurred during the animals’ light cycle.

### Systemic drug administration

Animals were injected with SCH 23390 (0.1 mg/kg, i.p.) or MK-801 (0.1 mg/kg, i.p.) dissolved in 0.9 % saline (0.1 mg/mL). These doses of SCH 23390 and MK-801 have previously been shown to impair fear conditioning and memory reconsolidation, respectively (Greba and Kokkinidis 2000; Inoue et al 2000; Lee et al. 2006). Vehicle-treated controls received injections of saline (1 mL/kg, i.p.).

### Systemic drug effects on contextual fear conditioning and memory testing

**Fig. 1:**
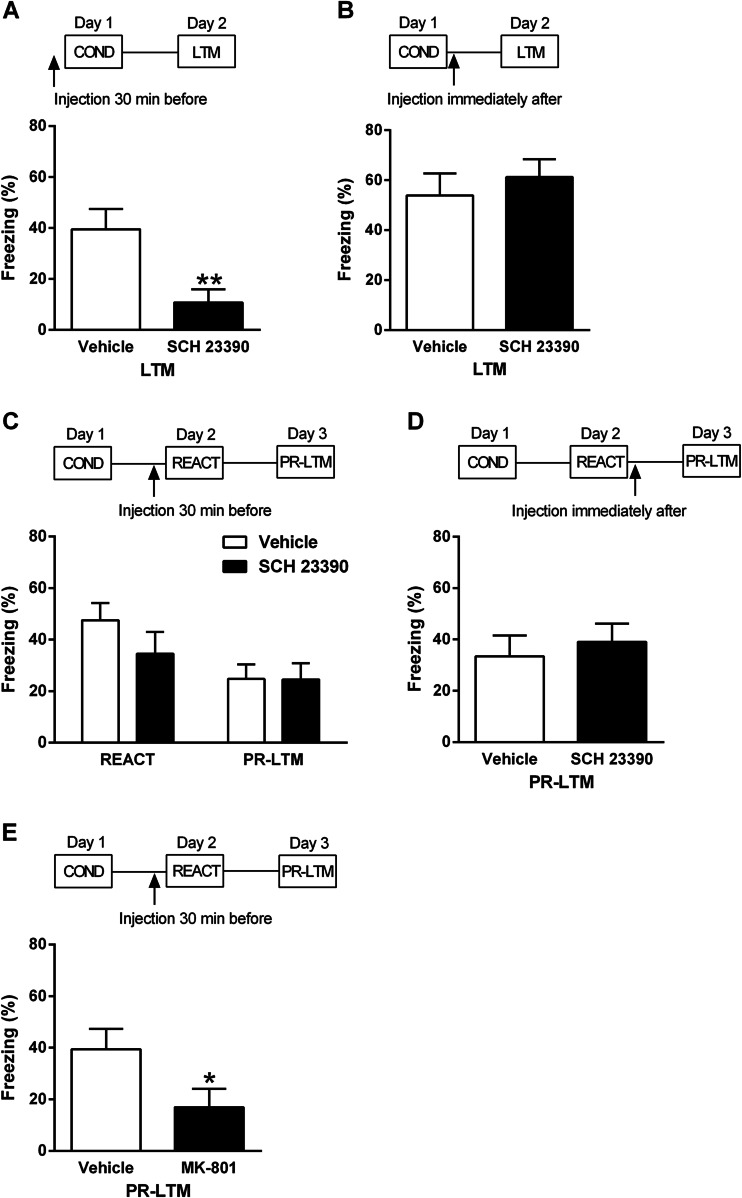
**a** Systemic SCH 23390 administration 30 min before contextual fear conditioning decreased freezing during LTM testing the next day (** *P* < 0.01). **b** SCH 23390 given immediately after conditioning had no effect on freezing during later LTM testing. **c** SCH 23390 given 30 min before brief memory reactivation (*REACT*) had no effects on freezing during REACT or during PR-LTM testing the next day. **d** SCH 23390 given immediately after REACT had no effect on freezing during later PR-LTM testing. **e** MK-801 given 30 min before REACT decreased freezing during PR-LTM testing the next day (* *P* < 0.05)

The effects of systemic SCH 23390 administration on different stages of contextual fear learning and memory processing were investigated using two chambers which have been described in detail elsewhere (Stevenson et al 2009). All animals underwent contextual fear conditioning on day 1 using testing parameters modified from our previous study (Stevenson 2011). Animals were conditioned in a novel context consisting of distinct visual (stripes or spots on two walls of the chambers with the house light on), auditory (60-dB white noise) and olfactory (ethanol cleaning solution) cues present during conditioning. The US used was mild electric shock delivered automatically through the floor bars of the chamber by a computer (MED-PC IV software, Med Associates, VT). The animals were placed in the chambers and after 2 min were subjected to four unsignalled shocks (0.5 mA, 0.5-s duration, 1-min inter-trial interval). The animals were removed from the chamber 2 min after the last shock and returned to the home cage. On day 2, all animals were returned to the conditioning chambers for 2 min. In some experiments, this served to test long-term memory (LTM), whereas in other experiments, this served to test memory retrieval and to reactivate memory before testing post-reactivation long-term memory (PR-LTM) for 2 min on day 3 (see Fig. 1). Separate cohorts of animals were injected with SCH 23390 or vehicle as follows: (1) 30 min before conditioning on day 1, (2) immediately after conditioning on day 1, (3) 30 min before reactivation on day 2, or (4) immediately after reactivation on day 2. Another cohort of animals was injected with MK-801 or vehicle 30 min before reactivation on day 2 to determine if the conditioning and reactivation parameters used allowed for the pharmacological disruption of reconsolidation; this NMDA receptor antagonist has previously been shown to disrupt the reconsolidation of other types of memory (Lee et al. 2006; Milton et al. 2008; Flavell and Lee 2013). The chamber floor bars and waste trays were cleaned with the ethanol cleaning solution between each session, and the animals were tested at approximately the same time of day on each day. Behaviour was recorded via digital cameras in the chamber ceilings for subsequent data analysis.

### Systemic drug effects on locomotor activity and shock sensitivity testing

To determine if any effects of systemic SCH 23390 given before conditioning on freezing during later LTM testing were due to non-specific effects on locomotion or nociception, some of the animals from the contextual fear conditioning and memory processing experiments described above were also used to examine the effects of this drug treatment on locomotor activity or shock sensitivity. Animals were tested 2–7 days after the last day of memory testing (Stevenson 2011).

Locomotor activity was tested in the open field using an apparatus (1 × 1-m base, 0.5-m high walls) made of black Perspex. Separate cohorts of animals were injected with SCH 23390 or vehicle as above, either 30 min or 24 h before testing. Animals were tested for 10 min in the open field. Testing occurred in a dimly lit room to match the lighting conditions present during contextual fear conditioning and memory testing. The floor of the open field was cleaned using a methanol solution after each testing session. Behaviour was digitally recorded for subsequent data analysis.

Shock sensitivity was tested using a modified version of a previously described paradigm (Quirk et al. 2000). A separate cohort of animals was injected with SCH 23390 or vehicle as above and tested 30 min later. Animals were placed in a novel chamber and after 10 min were subjected to 10 unsignalled shocks (0.5 s, 1-min inter-trial interval). The first shock was 0.05 mA and each subsequent shock increased in 0.05-mA increments, such that the last shock was 0.5 mA. The chamber floor bars and waste trays were cleaned with a methanol cleaning solution between each session. Behaviour during testing was digitally recorded for subsequent data analysis.

### Surgery

To determine the possible sites of action of SCH 23390 on contextual fear conditioning and locomotor activity, separate cohorts of experimentally naïve animals were implanted with cannulae targeting DH or BLA to infuse drug locally into these regions. Anaesthesia (isoflurane) and analgesia (buprenorphine) ensured complete inhibition of the hindpaw withdrawal reflex during surgery. Animals were placed in a stereotaxic frame and the incisor bar was adjusted to maintain the skull horizontal. For DH, guide cannulae (26-gauge; PlasticsOne, VA) were implanted bilaterally 0.5 mm dorsal to the target site using the following stereotaxic coordinates: 4.5 mm posterior and 3 mm lateral to bregma, and 3 mm ventral to the brain surface (Paxinos and Watson 1998). The stylets (33-gauge; PlasticsOne) extended 0.5 mm beyond the tip of the guide cannulae. These coordinates were based on our previous study investigating the effects of hippocampal SCH 23390 infusions on spatial learning and memory (Pezze and Bast 2012). For BLA, guide cannulae were implanted bilaterally 1 mm dorsal to the target site using the following stereotaxic coordinates: 2.8 mm posterior and 4.7 mm lateral to bregma, and 6.4 mm ventral to the brain surface. The stylets extended 1 mm beyond the tip of the guide cannulae. These coordinates were modified from our previous study investigating the effects of BLA inactivation on contextual fear memory expression (Stevenson 2011). Cannulae were secured with dental cement to four screws threaded into the skull. Animals were singly housed after surgery and given post-operative analgesia (buprenorphine and meloxicam). From 1–2 days after surgery, animals were mildly restrained every 1–2 days and the stylets were loosened and retightened to ensure that the cannulae remained unblocked; this also served to habituate the animals to handling during the central drug infusion procedure. Behavioural testing began 5–7 days after surgery.

### Central drug effects on contextual fear conditioning and locomotor activity

For DH infusions, 5 μg of SCH 23390 dissolved in 1 μL of 0.9 % saline was infused (Pezze and Bast 2012). For BLA infusions, 2.5 μg of SCH 23390 dissolved in 0.5 μL of 0.9 % saline was infused. This dose was adapted from our previous studies investigating the effects of BLA SCH 23390 infusion on acoustic startle regulation (Stevenson and Gratton 2004a, 2004b). The stylets were removed, and SCH 23390 or vehicle (0.9 % saline) was infused bilaterally at a rate of 0.5 μL/min using injectors (33-gauge; PlasticsOne), extending 0.5 mm (DH) or 1 mm (BLA) beyond the tips of the guide cannulae, which were connected to 1-μL Hamilton syringes via a length of polyethylene tubing. The injectors were left in place for 1 min following infusions before they were removed and the stylets replaced; behavioural testing started 10 min later. Animals were first given SCH 23390 or vehicle infusions before contextual fear conditioning using the same parameters as above except that the shock duration was increased to 1 s to reduce any potential deficit in freezing caused by surgery (Hart et al. 2009). The next day, the animals underwent LTM testing for 2 min as above. The same animals were then used 2–7 days later to test the effects of DH or BLA infusions of SCH 23390 or vehicle given 10 min before open field testing as above. Behaviour during LTM and open field testing was digitally recorded for subsequent data analysis.

### Histology

Upon completion of the central drug infusion experiments, the animals were deeply anaesthetized and transcardially perfused with 0.9 % saline followed by 4 % paraformaldehyde. Brains were removed and post-fixed in 4 % paraformaldehyde and kept at 4 °C until slicing. DH or BLA sections were obtained and stained for acetylcholinesterase as previously described (Stevenson et al. 2007).

### Data analysis

Freezing, defined as the absence of movement except for that related to respiration, was taken as a behavioural index of fear during LTM, reactivation and/or PR-LTM testing. Freezing behaviour was scored manually by one or two trained observers, one of whom was blind to the treatment group of the animal. Freezing was determined at 3-s intervals throughout testing, and the cumulative duration of freezing was then calculated and expressed as a percentage of each 2-min test. The effects of systemic SCH 23390 given before or after conditioning on freezing during LTM testing the next day were analysed separately using two-tailed unpaired *t* tests. The effects of systemic SCH 23390 given before reactivation on freezing during reactivation and PR-LTM testing the next day were analysed using a two-way analysis of variance (ANOVA), with treatment and test as the between- and within-subject factors, respectively. The effects of systemic SCH 23390 given after or MK-801 given before reactivation on freezing during PR-LTM testing the next day were analysed using separate two-tailed unpaired *t* tests. The effects of DH or BLA SCH 23390 given before conditioning on freezing at LTM test the next day were analysed separately using two-tailed unpaired *t* tests.

Behaviour in the open field was analysed using Ethovision software (Noldus, Netherlands). The total distance moved was determined and taken as an index of locomotor activity. The effects of systemic SCH 23390 given 30 min or 24 h before testing were analysed separately using two-tailed unpaired *t* tests. The effects of DH or BLA SCH 23390 given 10 min before testing were analysed in the same way.

Behaviour during shock sensitivity was scored manually to determine the threshold current which elicited ‘flinch’ and vocalization responses (Quirk et al. 2000). Behaviour was scored by two observers, one of whom was blind to the treatment group of the animal. The effects of systemic SCH 23390 given before testing were analysed using two-way ANOVA, with treatment and response as the between- and within-subject factors, respectively. All data are presented as the mean + SEM and the significance level for all comparisons was set at *P* < 0.05.

## Results

### Systemic SCH 23390 effects on contextual fear conditioning and memory processing

The effects of systemic SCH 23390 given 30 min before or immediately after contextual fear conditioning on freezing during LTM testing the next day are presented in Fig. 1a, b. Compared to vehicle (*n* = 10), SCH 23390 (*n* = 10) given before conditioning significantly decreased freezing at LTM test (t_(18)_ = 3.03, *P* < 0.01; Fig. 1a). No differences in freezing during LTM testing were observed between SCH 23390 (*n* = 10) and vehicle (*n* = 9) given after conditioning (t_(17)_ = 0.66, *P* > 0.1; Fig. 1b).

The effects of systemic SCH 23390 given 30 min before or immediately after memory reactivation on freezing during reactivation and during PR-LTM testing the next day are shown in Fig. 1c, d. There were no differences in freezing during reactivation or PR-LTM testing between SCH 23390 (*n* = 10) or vehicle (*n* = 10) given before reactivation (main effect of treatment: F_(1,18)_ = 0.67, *P* > 0.1; treatment × test interaction: F_(1,18)_ = 1.46, *P* > 0.1; Fig. 1c). Similarly, no differences in freezing during reactivation or PR-LTM testing were observed between SCH 23390 (*n* = 11) or vehicle (*n* = 11) given immediately after reactivation (main effect of treatment: F_(1,20)_ = 0.002, *P* > 0.1; treatment × test interaction: F_(1,20)_ = 2.82, *P* > 0.1; Fig. 1d).

The lack of effect of SCH 23390, given before or after reactivation, on freezing at PR-LTM test suggests that SCH 23390 did not affect memory reconsolidation. Various boundary conditions, such as memory strength and reactivation duration, are important in determining if a memory can undergo reconsolidation after its retrieval (Lee 2009). To test if the conditioning and reactivation parameters used allowed for memory reconsolidation to occur, we examined the effects of MK-801 given 30 min before reactivation on freezing during PR-LTM testing. Compared to vehicle (*n* = 9), MK-801 (*n* = 9) significantly decreased freezing at PR-LTM test (t_(16)_ = 2.13, *P* < 0.05; Fig. 1e). This result suggests that MK-801 impaired memory reconsolidation, in agreement with other studies (Lee et al. 2006; Milton et al. 2008; Flavell and Lee 2013). It also suggests that the lack of effect of SCH 23390 on reconsolidation was unlikely due to any boundary conditions associated with the parameters used.

### Systemic SCH 23390 effects on locomotor activity and shock sensitivity

The finding that SCH 23390, given before conditioning, reduced freezing at LTM test suggests that SCH 23390 impaired contextual fear conditioning. However, it is possible that SCH 23390 affected nociception during conditioning. Another possibility is that SCH 23390 had non-specific locomotor effects that endured until LTM testing the next day; similarly, the lack of effect of SCH 23390 given before reactivation on freezing at PR-LTM test the next day could also have been due to lasting drug effects on locomotor activity. We addressed these issues by examining the effects of systemic SCH 23390 on locomotor activity and shock sensitivity.

**Fig. 2:**
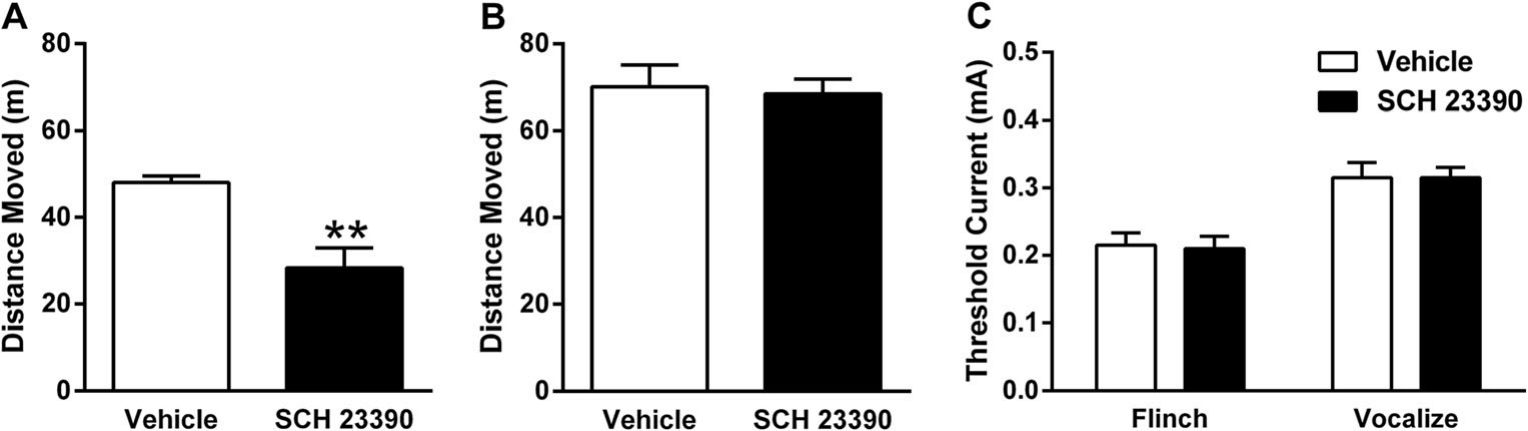
**a** Systemic SCH 23390 administration 30 min before open field testing decreased the distance moved during the test (** *P* < 0.01). **b** SCH 23390 given 24 h before the open field test had no effect on the distance moved during testing. **c** SCH 23390 had no effect on the threshold current required to elicit flinch or vocalization responses during shock sensitivity testing

The effects of SCH 23390 given 30 min before open field testing are presented in Fig. 2a. Compared to vehicle (*n* = 9), SCH 23390 (*n* = 11) significantly decreased locomotor activity (t_(18)_ = 3.69, *P* < 0.01). The effects of SCH 23390 given 24 h before open field testing are shown in Fig. 2b. In contrast to the effects of treatment 30 min before testing, no differences in locomotor activity were observed between SCH 23390 (*n* = 9) or vehicle (*n* = 9) when testing occurred 24 h after treatment (t_(16)_ = 0.27, *P* > 0.1). The effects of SCH 23390 given 30 min before shock sensitivity testing are depicted in Fig. 2c. There were no differences between SCH 23390 (*n* = 10) or vehicle (*n* = 10) treatment in the threshold current eliciting flinch or vocalization responses (main effect of treatment: F_(1,18)_ = 0.015, *P* > 0.1; treatment × response interaction: F_(1,18)_ = 0.023, *P* > 0.1). These results likely rule out the possibility that SCH 23390 given before conditioning reduced freezing at LTM test due to non-specific drug effects on locomotion or nociception, which generally agrees with previous findings (Bruhwyler et al. 1991; Inoue et al. 2000).

### Central SCH 23390 effects on contextual fear conditioning and locomotor activity

**Fig. 3:**
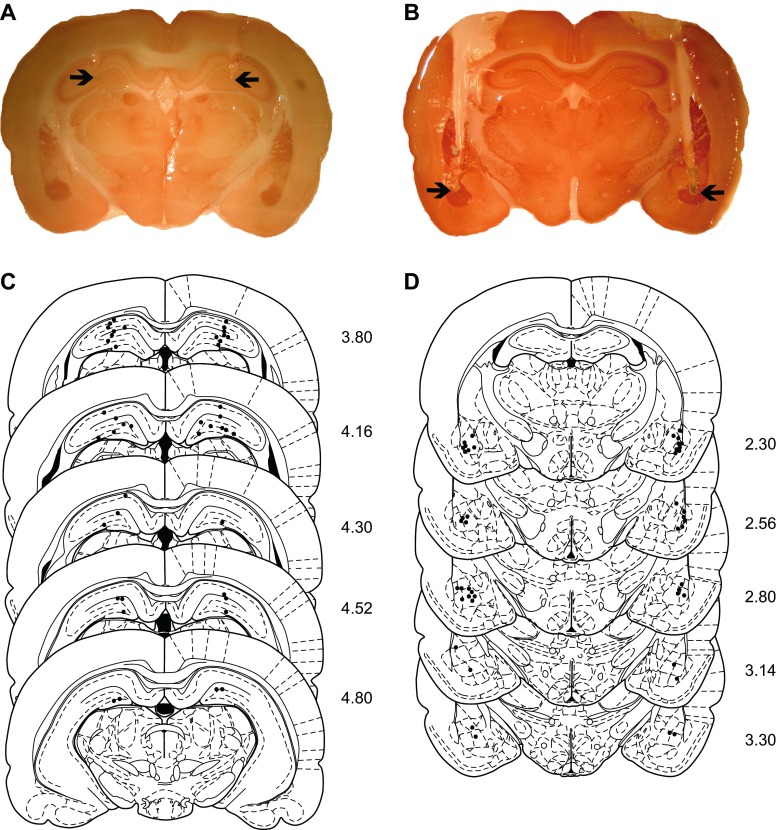
Representative cannulae placements in **a** DH and **b** BLA are indicated by the *arrows*. Schematic representation of cannulae placements in **c** DH and **d** BLA indicated by the *dots* (distance (mm) posterior to bregma is indicated to the *right* of each section) is presented

**Fig. 4:**
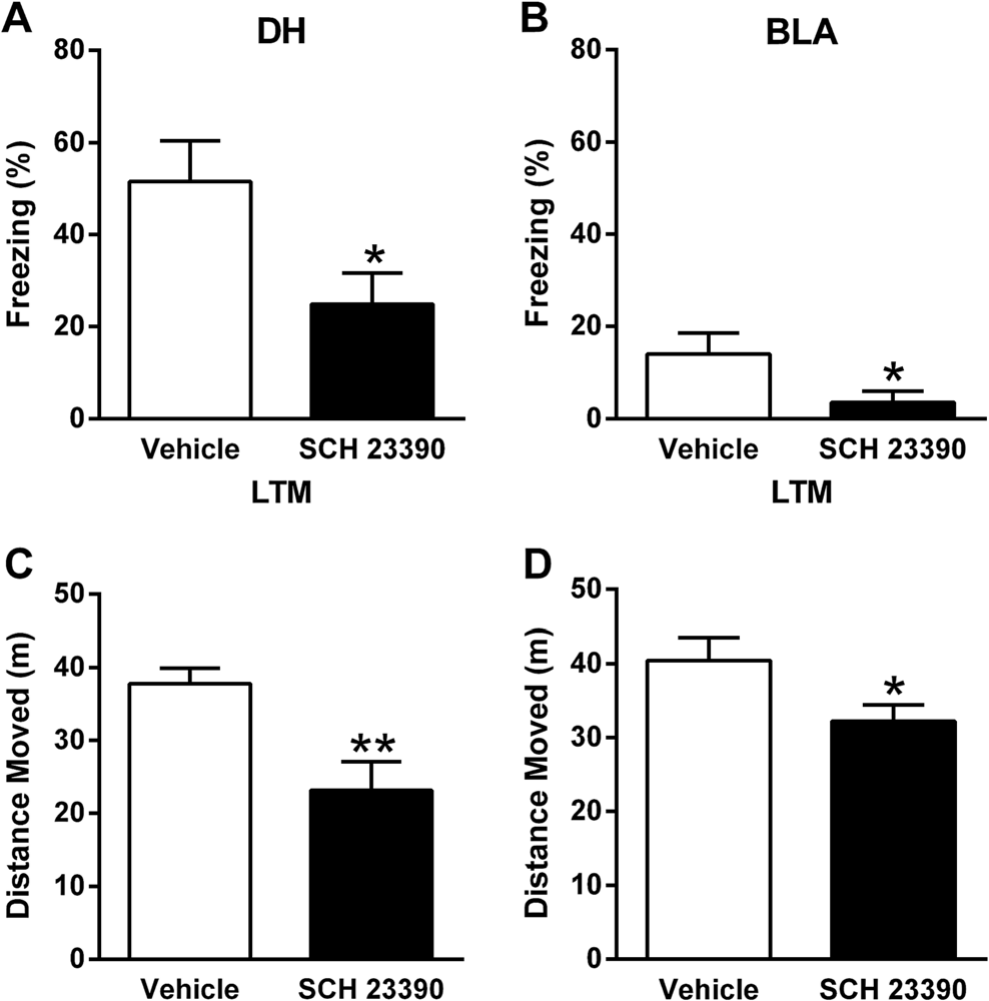
**a** Infusing SCH 23390 into DH 10 min before contextual fear conditioning decreased freezing during LTM testing the next day (* *P* < 0.05). **b** SCH 23390 infusion into BLA 10 min prior to conditioning also decreased freezing at LTM test (* *P* < 0.05). **c** Intra-DH infusion of SCH 23390 10 min before testing decreased the distance moved in the open field (** *P* < 0.01). **d** Intra-BLA SCH 23390 infusion 10 min before the test also decreased the distance moved in the open field (* *P* < 0.05)

To determine if SCH 23390 acts in DH and/or BLA in mediating its effects on contextual fear conditioning, we examined the effects of local drug infusion into these regions. Only data from animals with histologically confirmed cannulae placements in DH or BLA (i.e. lateral or basal amygdaloid nuclei) were included in the analysis (Fig. 3). The effects of infusing SCH 23390 into DH 10 min before contextual fear conditioning on freezing during LTM testing the next day are presented in Fig. 4a. Compared to vehicle (*n* = 10), infusion of SCH 23390 (*n* = 12) into DH before conditioning significantly decreased freezing at LTM test (t_(20)_ = 2.44, *P* < 0.05). The effects of intra-BLA infusion of SCH 23390 10 min before conditioning on freezing during later LTM testing are shown in Fig. 4b. Freezing was markedly decreased in animals with BLA cannulae, possibly due to partial BLA damage caused by the implants which has been reported previously (Fendt 2001). Nevertheless, compared to vehicle (*n* = 10), SCH 23390 (*n* = 12) infused into BLA before conditioning also significantly decreased freezing at LTM test (t_(20)_ = 2.10, *P* < 0.05). These results suggest that SCH 23390 impairs contextual fear conditioning, at least in part, by acting in DH and BLA.

We also examined the effects of infusing SCH 23390 into DH or BLA on locomotor activity to determine if the acute effects of systemic administration reported above (see Fig. 2a) involve these regions. The effects of SCH 23390 infusion into DH 10 min before open field testing are presented in Fig. 4c. Compared to vehicle (*n* = 10), intra-DH infusion of SCH 23390 (*n* = 12) significantly decreased locomotor activity (t_(20)_ = 3.08, *P* < 0.01). The effects of BLA SCH 23390 infusion 10 min before the open field test are shown in Fig. 4d. Compared to vehicle (*n* = 9), intra-BLA SCH 23390 (*n* = 14) resulted in a small but significant decrease in locomotor activity (t_(21)_ = 2.22, *P* < 0.05). These results suggest that DH and BLA are sites of action for the inhibitory effects of SCH 23390 on locomotor activity, broadly confirming previous findings (McGregor and Roberts 1993; Pezze and Bast 2012).

## Discussion

This study investigated the effects of the dopamine D1/D5 receptor antagonist SCH 23390 on contextual fear memory processing. We found that SCH 23390 impaired the acquisition, but not the consolidation, retrieval or reconsolidation, of contextual fear memory. The lack of effect of SCH 23390 on reconsolidation was unlikely due to any boundary conditions associated with the behavioural parameters used, given that the NMDA receptor antagonist MK-801 was found to impair memory reconsolidation using these parameters. We found that SCH 23390 decreased locomotor activity when given 30 min, but not 24 h, after administration and that it had no effect on shock sensitivity. This indicates that the acquisition impairment caused by SCH 23390 was unlikely due to non-specific drug effects on nociception during conditioning or on locomotion during memory testing the next day. SCH 23390 infused locally into DH or BLA also impaired conditioning and decreased locomotor activity, indicating that it mediates its effects, at least in part, by acting in these regions.

The finding that systemic SCH 23390 administration impaired contextual fear conditioning, but not memory consolidation or retrieval, is congruent with a previous report (Inoue et al. 2000). It is possible that we would have observed a drug effect on memory consolidation if a longer-duration LTM test had been used. However, Inoue et al. (2000) reported a similar negative finding of SCH 23390 on consolidation using a longer duration LTM test. Moreover, we did observe a drug effect on acquisition using the same brief duration LTM test that we used in the consolidation experiments. The lack of effect of SCH 23390 on shock sensitivity has been shown previously (Inoue et al. 2000) and suggests that it did not affect somatosensory perception of the US during conditioning. The acute locomotor effect of SCH 23390 agrees with previous findings (Bruhwyler et al. 1991), and its lack of effect 24 h after administration is in keeping with the brief (~25 min) half-life of this drug in rats (Bourne 2001). This suggests that SCH 23390 given before conditioning did not have non-specific locomotor effects during memory testing the next day. Taken together, these findings suggest that D1-like receptor signalling at the time of conditioning is involved in encoding contextual fear.

It is worth noting that SCH 23390 can modulate the consolidation of contextual fear memory under certain conditions. Intra-DH infusion of SCH 23390 after conditioning attenuates the facilitatory effects of corticosterone on contextual fear encoding (Liao et al. 2013). Male rats exposed to females after conditioning show impaired contextual fear retention, and this effect is blocked by infusion of SCH 23390 into DH post-conditioning (Bai et al. 2009). However, in both of these studies, SCH 23390 had no effect in the respective control conditions, suggesting that D1/D5 receptor signalling in DH is not necessary for contextual fear memory consolidation. The lack of effect of systemic SCH 23390 administration on the retrieval of contextual fear memory is more difficult to interpret given its acute locomotor effects. It is possible that SCH 23390 impaired retrieval, which would have reduced freezing, but that this effect was masked by the locomotor activity impairing effects of the drug, which might have resembled enhanced freezing. In contrast, central infusion studies have shown a role for D1-like receptor signalling in learned fear retrieval. Intra-BLA infusion of SCH 23390 impairs the retrieval of fear-potentiated startle and second-order auditory fear memory (Lamont and Kokkinidis 1998; Nader and LeDoux 1999), whereas infusion into nucleus accumbens shell enhances contextual fear retrieval (Albrechet-Souza et al. 2013). Therefore, studies investigating the effects of central SCH 23390 infusions on the retrieval of contextual fear memory might be warranted.

Contrary to the present results, previous studies have shown that SCH 23390 disrupts memory reconsolidation in other paradigms. SCH 23390 impairs the reconsolidation of passive avoidance memory (Sherry et al. 2005), albeit at a greater dose (0.5 mg/kg) than the one used here. Therefore, it is possible that we may have observed an effect had we used a greater dose of drug. However, SCH 23390 given at a similar dose (0.08 mg/kg) to that used in the present study has been found to disrupt cocaine-associated memory reconsolidation (Yan et al. 2014). Another possibility is that certain boundary conditions related to the behavioural parameters used here did not allow for the impairment of memory reconsolidation by SCH 23390. For example, previous studies have shown that using strong conditioning or brief reactivation parameters can result in resistance to the pharmacological disruption of memory reconsolidation (Bustos et al. 2009; Lee and Flavell 2014). Memory updating during the reconsolidation process is thought to involve a prediction error signal to incorporate new information into the existing memory (Lee 2009). Thus, it is possible that the reactivation duration used in the present study was too brief to engage prediction error signalling and render the memory amenable to disruption. To address this issue, we also examined the effects of MK-801 on memory reconsolidation using the same testing parameters that were used in the SCH 23390 experiments. We found that MK-801 impaired memory reconsolidation, confirming previous results (Lee et al. 2006; Milton et al. 2008; Flavell and Lee 2013). This indicates that the lack of effect of SCH 23390 on the reconsolidation of contextual fear memory was unlikely due to any boundary conditions surrounding the parameters used.

Another possibility is that D1-like receptor signalling is involved in the destabilization of contextual fear memory. During retrieval, memory can become labile by undergoing destabilization before later becoming restabilized through reconsolidation. This process is thought to play an important role in maintaining memory persistence and relevance following memory updating (Lee 2009). Evidence indicates that memory retrieval and destabilization are dissociable processes (Ben Mamou et al. 2006; Milton et al. 2013), therefore, the lack of effect of SCH 23390 on retrieval reported here does not preclude a role for D1 receptor signalling in mediating destabilization. Recent studies have shown that dopamine transmission is involved in memory destabilization. Inhibition of dopamine cell activity in the VTA before appetitive memory retrieval prevents the disruptive effects of post-retrieval MK-801 on reconsolidation (Reichelt et al. 2013). SCH 23390 infused into DH before the retrieval of object recognition memory also mitigates the reconsolidation impairing effects of anisomycin infusion after retrieval (Rossato et al. 2014). These results suggest that memory updating through prediction error signalling, during which dopamine transmission plays a crucial role (Schultz 2013), involves the initial destabilization process. Furthermore, D1-like receptor signalling might also be involved in the destabilization of contextual fear memory.

We found that infusing SCH 23390 into DH or BLA impaired contextual fear conditioning. To our knowledge, this has not been shown previously. However, this result broadly agrees with other findings showing that D1-like receptor signalling in these regions is involved in synaptic plasticity and, more specifically, plays a role in aversive learning involving contextual cues. SCH 23390 impairs long-term potentiation in DH and BLA (Otmakhova and Lisman 1996; Huang and Kandel 1995, 2007). Mice lacking D1 receptors in dentate gyrus granule cells show impaired contextual fear conditioning (Sariñana et al. 2014). These mice also exhibit similar fear levels in the conditioning context and another separate context after learning, suggesting that they show generalization of contextual fear. In the present study, we did not examine the effects of SCH 23390 on contextual fear generalization, although this could be investigated in the future. Intra-BLA infusion of SCH 23390 impairs conditioned place avoidance learning (Macedo et al. 2007). Infusing SCH 23390 into the central nucleus of the amygdala (CE) before auditory fear conditioning results in reduced freezing at memory test, in response to tones and during the interval between tone presentations, indicating impaired conditioning to auditory and background contextual cues (Guarraci et al. 1999). Interestingly, D1-like receptor expression in CE is low relative to the adjacent BLA (Scibilia et al. 1992), suggesting that the effects of intra-CE infusion of SCH 23390 reported by Guarraci et al. (1999) might instead be mediated by BLA. Indeed, infusion of a D1/D5 receptor agonist into BLA enhances contextual fear conditioning under certain conditions (Biedenkapp and Rudy 2008).

How exactly D1-like receptor signalling in DH and BLA is involved in encoding contextual fear memory remains to be determined. During contextual fear conditioning, a spatial representation of the context is encoded and this representation becomes associated with the US (Anagnostaras et al. 2001). Previous studies have shown that intra-DH infusion of SCH 23390 impairs spatial learning (O’Carroll et al. 2006; Pezze and Bast 2012). This raises the possibility that SCH 23390 infused into DH may impair contextual fear conditioning by interfering with the encoding of the spatial representation of the context. For example, D1-like receptor signalling might be involved in encoding the various contextual elements into a distinct configural representation of the context (Rudy and Sutherland 1995). Although a previous study found that amphetamine did not impair configural associations during appetitive learning (Blackburn and Hevenor 1996), this issue has received little scrutiny to date and future studies could investigate the effects of SCH 23390 on configural learning. Another possibility is that SCH 23390 disrupts attentional mechanisms involved in contextual encoding by DH, and evidence from studies on spatial and appetitive learning supports this idea (Muzzio et al. 2009). Alternatively, evidence also indicates a role for DH in associating the context and US during contextual fear learning (Chang et al. 2008; Lee 2010), and D1-like receptor signalling could be involved in this process. Similarly, intra-BLA SCH 23390 infusion may disrupt contextual fear conditioning by interfering with encoding the context-US association. Previous studies showing that amygdala SCH 23390 infusion impairs aversive learning lend support to this idea (Guarraci et al. 1999; Greba and Kokkinidis 2000). Further work is needed to determine the respective roles of DH and BLA D1-like receptor signalling in modulating contextual fear conditioning.

In summary, we have confirmed previous findings demonstrating that SCH 23390 impairs the acquisition of contextual fear and extended them by showing that this effect is mediated partly by DH and BLA. However, we cannot rule out the involvement of other regions in mediating the effects of SCH 23390 on contextual fear conditioning. Through their inter-connections with DH and BLA, corticostriatal areas such as the medial prefrontal cortex, dorsal striatum and nucleus accumbens form part of a wider neural circuit involved in contextual fear processing (Haralambous and Westbrook 1999; Levita et al. 2002; White and Salinas 2003; Rozeske et al. 2014). These areas receive dopamine input from VTA (Lammel et al. 2014), and recent evidence indicates that D1 receptor signalling in these regions regulates contextual fear conditioning (Ikegami et al. 2014). Our results add to a growing body of evidence indicating that contextual fear processing is modulated by both D1- and D2-like receptor signalling (Biojone et al. 2011; de Souza Caetano et al. 2013; Colombo et al. 2013; Wen et al. 2014).
